# Maternal Lifestyle Factors Affecting Breast Milk Composition and Infant Health: A Systematic Review

**DOI:** 10.3390/nu17010062

**Published:** 2024-12-27

**Authors:** Giuliana Favara, Andrea Maugeri, Martina Barchitta, Erika Lanza, Roberta Magnano San Lio, Antonella Agodi

**Affiliations:** Department of Medical and Surgical Sciences and Advanced Technologies “GF Ingrassia”, University of Catania, 95123 Catania, Italy; giuliana.favara@unict.it (G.F.); andrea.maugeri@unict.it (A.M.); martina.barchitta@unict.it (M.B.); lanzaerika18@gmail.com (E.L.); robertamagnanosanlio@unict.it (R.M.S.L.)

**Keywords:** maternal nutrition, pregnancy, lifestyles, breast milk, infant health, infant outcomes

## Abstract

Background/Objectives: Breast milk is a dynamic, personalized nutrition source, influenced by maternal diet, lifestyle, and environmental factors, which shape its composition and impact infant health. This review synthesizes evidence on the associations between maternal lifestyles (e.g., diet, physical activity, smoking), breast milk composition, and child health, offering insights for interventions to optimize breastfeeding benefits. Methods: We searched Web of Science, Medline, Embase, and PubMed for studies published up to March 2024 using predefined terms. Results: Out of 5244 articles, 20 studies met the inclusion criteria. Maternal Body Mass Index and macronutrient intake significantly affected breast milk fatty acid composition, influencing infant growth, cognitive development, and metabolic health. Micronutrient intake, particularly iodine, omega-3 fatty acids, and vitamins, was linked to better neurodevelopment and reduced atopic risks. Maternal diet and supplementation improved breast milk nutrient profiles and infant outcomes, though exposure to toxins like ochratoxin A raised concerns. Smoking was associated with altered milk composition, including lower osteopontin levels, potentially affecting infant immunity and growth. Conclusions: This review emphasizes that adequate intake of key nutrients is essential for infant development, highlighting the need for policies that address nutritional deficiencies, promote healthy lifestyles, and reduce socio-economic barriers. These efforts can improve outcomes for both mothers and children, enhancing public health and reducing disparities.

## 1. Introduction

Breast milk is widely recognized as the optimal source of nutrition for infants during the first months of life, providing essential energy, bioactive compounds, and fatty acids necessary for the growth and development of full-term infants [1,2]. The World Health Organization (WHO) recommends exclusive breastfeeding within the first hour of birth and continuing for at least six months to promote optimal growth, development, and overall health [3,4]. These recommendations underscore the critical role of breastfeeding in global nutritional policies aimed at improving maternal and child health [5,6].

The composition of human milk is remarkably dynamic, undergoing continuous changes during feeding, throughout the day, over the course of lactation, and among individuals and populations. This adaptive quality ensures that breast milk remains a responsive and personalized source of nourishment for each infant [7]. For example, the lipid content and composition vary significantly, reflecting not only inter-individual differences but also changes within the same individual over time [1,7]. Such variability is heavily influenced by maternal factors, which play a critical role in determining the quality and composition of breast milk, highlighting the potential for targeted interventions to optimize the nutritional benefits of breastfeeding [8]. This unique adaptability results from a combination of intrinsic biological mechanisms and external factors, including maternal genetics, dietary habits, lifestyle choices, and environmental exposures [9,10].

Among these, maternal nutrition plays a pivotal role in shaping the nutritional and immunological profile of breast milk through complex metabolic pathways that exert both direct and indirect effects. Nutrients that a mother consumes, such as fatty acids, vitamins, and other bioactive compounds, not only influence the nutrient content of the milk but also modulate its quality, determining how effectively it supports infant growth, development, and immune function [11,12]. For instance, levels of fatty acids and fat-soluble vitamins such as vitamins A, C, B6, and B12 are directly correlated with maternal dietary intake, which means they can vary according to the mother’s food choices and nutritional needs [13]. At the same time, breastfeeding, while offering substantial health benefits, can also serve as a vector for exposure to environmental contaminants [14]. Infants consuming large quantities of breast milk may ingest measurable levels of xenobiotics, including chemical pollutants such as organochlorine pesticides and dioxins, which have raised concerns regarding their potential adverse effects on infant health and development [15,16,17].

In this context, understanding the multifaceted interactions between maternal lifestyle factors, breast milk composition, and infant health outcomes is critical for informing effective nutritional policies and educational strategies. This systematic review aims to provide an updated synthesis regarding the association between maternal diet and lifestyles, human milk composition, and child outcomes, offering actionable insights to guide interventions that maximize the health benefits of breastfeeding.

## 2. Materials and Methods

### 2.1. Literature Search

From its inception until March 2024, we conducted an extensive literature search across Web of Science, Medline, Embase, and PubMed. The primary aim of this search was to identify epidemiological studies that examined the influence of maternal nutrition and lifestyle on human milk composition and child health outcomes. Two authors (G.F. and A.M.) independently performed the search using the following terms: (“breast milk” OR “human milk” OR “maternal milk”) AND (diet OR nutrition OR smoking OR “tobacco smoke” OR “physical activit”) AND (pregnan OR gestation*). The methodology employed in this systematic review followed the guidelines outlined in the Preferred Reporting Items for Systematic Reviews and Meta-analyses (PRISMA) statement, along with the recommendations provided in the Cochrane Handbook [18]. The PRISMA checklist is available as a Supplementary file.

### 2.2. Selection Criteria and Data Extraction

The authors independently reviewed and selected articles based on the following criteria: (i) published in English; (ii) observational epidemiological studies; (iii) focused on pregnant and/or breastfeeding women and/or their offspring; (iv) investigating how maternal lifestyle factors—such as diet, physical activity, and smoking—impact the qualitative and quantitative composition of breast milk; and (v) assessing potential associations with infant health outcomes.

The exclusion criteria were as follows: (i) studies involving animals or conducted in veterinary settings; (ii) studies solely examining the impact of breast milk composition on infant health outcomes without addressing the influence of maternal lifestyle; (iii) abstracts without full-text availability or articles not published in English; (iv) case reports, case series, comments, letters, editorials, and reviews; and (v) unpublished studies or research using in vitro or animal models.

For each eligible study, the authors independently extracted key data using a structured format, including the first author’s last name, year of publication, country, study population, maternal lifestyle factors, and infant outcomes examined. Neonatal outcomes included anthropometric measures, preterm birth, neurological development, growth, cancer, renal diseases, atopic conditions, and allergies. Any discrepancies between G.F. and A.M. during the selection and data extraction processes were resolved through consultation with a third author (A.A.).

## 3. Results

### 3.1. Study Selection and Characteristics of Included Studies

**Figure 1:**
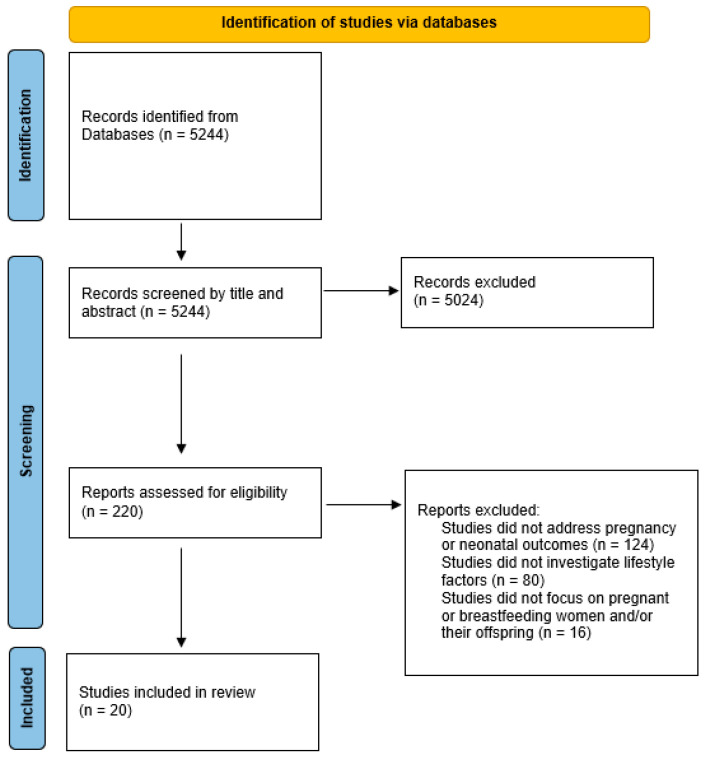
PRISMA flow diagram of study selection.

Figure 1 presents the study selection process following the PRISMA flow diagram. After the removal of duplicates, 5244 unique articles were identified across the databases. Of these, 5024 were excluded during the title and abstract screening phase. Next, 220 studies were excluded after a full-text review based on the selection criteria: 124 studies did not address pregnancy or neonatal outcomes, 80 studies did not investigate lifestyle factors, and 16 studies did not focus on pregnant or breastfeeding women and/or their offspring.

Accordingly, a total of 20 studies, published between 2005 and 2023, were selected for inclusion. These studies were conducted in various countries, including Turkey (*n* = 4), Australia (*n* = 2), the United States (*n* = 2), Sweden (*n* = 2), and one study each from Spain, Italy, Finland, Iran, Austria, Sudan, Peru, Norway, China, and Morocco. All included studies focused on pregnant and/or breastfeeding women and their children. Among these studies, 17 evaluated the effects of diet and nutritional status (Table 1), three examined the impact of smoking (Table 2), and none investigated the effects of physical activity. The following paragraphs summarize the evidence produced by each study, organized coherently by the type of exposure assessed.

#### 3.1.1. Impact of Maternal Body Mass Index and Macronutrients Intake

Several studies have highlighted the significant influence of maternal pre-pregnancy Body Mass Index (BMI) and dietary intake on the fatty acid composition of breast milk, which in turn affects infant growth and development (Table 1). De la Garza Puentes and colleagues demonstrated that maternal pre-pregnancy Body Mass Index (BMI) can influence fatty acids (FAs) in breast milk, as well as infant growth and cognition. Breast milk from overweight or obese mothers showed an increase in saturated fatty acids and a higher *n*-6 ratio, with reduced levels of alpha-linolenic acid (ALA), DHA, and monounsaturated fatty acids. At six months of age, infants’ BMI-for-age was inversely associated with arachidonic acid (AA) and docosahexaenoic acid (DHA) in colostrum but positively associated with the *n*-6 ratio. Infant cognitive development was positively influenced by the presence of linoleic acid, *n*-6 PUFAs, ALA, and DHA [19]. The study by Tekin-Guler and colleagues examined how pre-pregnancy obesity and maternal diet influenced the fatty acid profile in breast milk and its effects on infant growth. Milk from obese mothers had lower levels of ALA, DHA, ARA, and total *n*-3 fatty acids compared to that from normal-weight mothers. Interestingly, there was a positive association between ARA in foremilk and weight-for-age percentile, suggesting that higher levels of ARA in foremilk are associated with better growth in infants [20]. The study conducted by Nyuar and colleagues investigated the fatty acid composition of colostrum, transitional, and mature milk from Northern Sudanese women, whose traditional diet is high in carbohydrates and low in fat. While the arachidonic acid levels in the milk samples were comparable to international averages, the DHA content was notably low. As a result, the study indicates that breast milk from mothers with such a diet is unlikely to provide sufficient DHA to support optimal postnatal neuro-visual development [21]. The research by Binder and colleagues emphasizes the importance of maternal nutrition and BMI in shaping breast milk composition to support optimal growth and neurodevelopment, particularly in preterm infants. The study highlights the significant influence of maternal diet and BMI on the composition of human milk, particularly its macronutrients and bioactive hormones. Increased maternal protein intake was associated with higher levels of protein, fat, carbohydrates, and energy in breast milk, as well as elevated concentrations of adiponectin and leptin—two hormones essential for infant growth and metabolic health. Additionally, maternal BMI was positively linked to both dietary protein intake and protein levels in breast milk [22]. Similarly, the study conducted by Bahreynian and colleagues examined the impact of maternal dietary fat intake on the composition of breast milk and infant growth during the first year. Their findings revealed an inverse relationship between maternal fat intake and eicosapentaenoic acid (EPA) levels in breast milk, which was associated with lower infant weight at four months. Additionally, an inverse association between maternal dietary fat intake and docosahexaenoic acid (DHA) levels in breast milk was observed, impacting infant weight at 12 months [23].

#### 3.1.2. Effects of Maternal Micronutrient Intake, Dietary Deficiencies, and Supplementations

Other studies have investigated the impact of maternal micronutrient intake and supplementation on breast milk composition and infant health outcomes, emphasizing the crucial role of adequate maternal nutrition in supporting optimal infant development. Hoppu and colleagues investigated the impact of maternal intake of vitamins C and E, through both diet and supplementation, on the antioxidant composition of breast milk and its potential to reduce the risk of atopy in infants. The results show that maternal dietary intake of vitamin C, rather than supplementation, significantly influenced the concentration of vitamin C in breast milk. A higher concentration of vitamin C was associated with a reduced risk of atopy in infants, especially in those whose mothers had food hypersensitivity [24]. Similarly, Han and colleagues underscore the significant impact of maternal selenium (Se) intake on both human milk composition and child outcomes. The study highlights that inadequate maternal Se levels are associated with postpartum weight retention (PPWR) and suboptimal infant growth outcomes, such as risks of underweight and overweight [25]. The study by Næss and colleagues highlights that breastfed infants in Norway are at risk of insufficient iodine intake during the first months of life. Maternal iodine status and breast milk iodine concentration were positively associated with infant iodine levels. However, breastfed infants had significantly lower urinary iodine concentrations (UICs) compared to formula-fed infants at both 3 and 6 months. No association was found between iodine levels and infant thyroid function [26]. As shown in the study by Stinca and colleagues, maternal iodine deficiency during pregnancy and breastfeeding significantly reduces the iodine available to infants through breast milk. This deficiency increases the risk of thyroid dysfunction and developmental issues in infants, including potential long-term cognitive impairments linked to iodine-deficiency-induced hypothyroidism, which affects critical brain development processes [27].

More specifically, some research on maternal supplementation has shown positive effects on breast milk composition and subsequent infant health outcomes. The study by Finkelstein and colleagues highlighted the role of maternal iron status during pregnancy in infant iron absorption and growth. While prenatal iron supplementation had no direct impact on infant iron levels or breast milk composition, higher fetal iron exposure was associated with better infant growth, particularly in length. Additionally, infant iron absorption was influenced by higher levels of transferrin receptor and copper [28]. Warstedt and colleagues highlighted that maternal omega-3 supplementation during pregnancy and breastfeeding increases levels of omega-3 fatty acids, EPA, and DHA in breast milk, which are linked to a lower incidence of IgE-associated allergic diseases in infants [29]. Similarly, Dunstan and colleagues found that fish oil supplementation increased DHA and EPA levels in breast milk, positively associating with better infant neurological development, particularly in eye–hand coordination [30]. Probiotic supplementation studies, such as those by Böttcher and colleagues, showed that *Lactobacillus reuteri* reduces TGF-β2 levels in colostrum, lowering the risk of sensitization and IgE-associated eczema in infants [31]. Boyle and colleagues explored prenatal supplementation with *Lactobacillus rhamnosus* GG (LGG), finding that while the treatment altered immune-related components in breast milk, there was no significant reduction in the risk of eczema in infants [32].

#### 3.1.3. Influence of Other Foods or Dietary Factors

Some studies have examined how maternal dietary choices, along with socio-economic factors, influence breast milk composition and potentially impact infant health outcomes.

The research conducted by Lutter and colleagues suggests that maternal egg consumption during lactation enhances the nutrient composition of breast milk, potentially supporting infant development. The study also highlights that egg consumption, providing essential nutrients like choline, is associated with socio-economic factors: wealthier women consume more eggs, which may lead to better nutritional outcomes in their breast milk. Economic barriers, more than cultural factors, appear to limit egg consumption, particularly in lower-income groups, which could impact the nutritional quality of breast milk and infant health [33].

One such study investigated the relationship between maternal dietary habits and the presence of ochratoxin A (OTA) in breast milk and cord blood. The study by Biasucci and colleagues found that dietary intakes were linked to higher levels of OTA in maternal serum, which then correlated with increased concentrations of OTA in breast milk. While the study did not find direct effects on infant health outcomes, such as weight or development, it suggested that prolonged exposure to such toxins via breast milk could have implications for the infant’s health [34].

The study by Henning and colleagues examined the effects of pomegranate juice consumption on breast milk and infant health. Specifically, pomegranate juice contains ellagitannins, which are metabolized into ellagic acid and urolithins—compounds with neuroprotective, antioxidant, and anti-inflammatory benefits. The study finds that these beneficial metabolites, including urolithin A-glucuronide (UAG) and urolithin B-glucuronide (UBG), are passed to infants through breast milk and detected in their urine. Additionally, maternal pomegranate juice intake alters the breast milk’s microbiota, with significant changes such as a reduction in *Lactococcus* and *Subdoligranulum* and an increase in *Firmicutes/Faecalibacterium*. These modifications in breast milk’s microbiota positively influence the infant’s gut microbiota, promoting the growth of beneficial genera such as *Lachnoclostridium* and *Staphylococcus*, and contributing to better overall health [35].

### 3.2. Effects of Maternal Smoking Habits

Three studies examined the effects of maternal smoking on the composition of breast milk and maternal–infant outcomes. One study by Aksan and colleagues highlighted the importance of osteopontin (OPN), a glycosylated phosphoprotein in breast milk, which is crucial for lactation and immune system development. The study found that smoking mothers had significantly lower OPN levels in their breast milk compared to non-smoking mothers throughout pre-pregnancy, pregnancy, and lactation. Infants of mothers with higher OPN levels showed better growth outcomes, including higher weight and length at one and three months of age. Furthermore, lower OPN levels in breast milk were linked to an increased risk of infections and autoimmune diseases in infants [36]. Yalçın and colleagues conducted a study assessing maternal factors influencing mercury (Hg) concentrations in breast milk and their effects on infant growth and development. The study found that both active and passive cigarette smoke increased mercury concentrations in breast milk. Despite this, the study indicated that mercury levels measured 10 to 20 days postpartum had no significant impact on the weight, height, or head circumference of infants who were exclusively breastfed at five months and two years of age. Furthermore, all infants demonstrated normal development, regardless of the mercury concentrations in their mothers’ breast milk [37]. The study conducted by Ozkan and colleagues investigated the effects of maternal smoking on leptin levels in breast milk and its impact on neonatal health. The findings revealed that smoking during pregnancy did not significantly alter leptin levels in breast milk. However, a significant decrease in neonatal serum leptin concentrations was observed in infants born to smoking mothers compared to those born to non-smoking mothers [38].

## 4. Discussion

Breastfeeding is a vital intervention to reduce child mortality and non-communicable diseases (NCDs) later in life, yet it remains underutilized. Only about 50% of newborns begin breastfeeding within the first hour of life, and just 67% are exclusively breastfed during the first two days [39,40]. Moreover, breast milk plays a crucial role in shaping an infant’s metabolic and immune systems, with lasting effects on their long-term health outcomes [4]. However, the composition of breast milk is influenced by various maternal factors, such as underlying health conditions (e.g., obesity, allergies, or celiac disease), dietary habits, and lifestyle choices.

It is widely recognized that a healthy lifestyle during the periconceptional, prenatal, and postnatal periods is crucial for promoting both maternal and infant health, while also reducing the risk of NCDs throughout life [39,40]. Other important factors include the method of delivery, antibiotic use, gestational age, and the stage of lactation, all of which can influence the microbiota of the milk and consequently infant health [41,42,43,44,45].

Given the complexity of these interactions, our systematic review seeks to explore the critical relationship between maternal lifestyles, breast milk composition, and infant health outcomes. By shedding light on these connections, we aim to highlight the importance of targeted nutritional policies and educational strategies to promote optimal health for both mothers and their children.

First of all, our work highlights the significance of maternal diet as a modifiable factor that directly influences the composition of breast milk composition and the long-term health outcomes of infants, also suggesting that a mother’s diet is a powerful determinant of her child’s early development and overall health [46].

A key takeaway from our analysis is the profound influence of maternal obesity and pre-pregnancy BMI on the fatty acid profile of breast milk. Overweight and obese mothers consistently show higher levels of saturated fatty acids and an imbalanced *n*-6/*n*-3 ratio in their milk, which can negatively impact infant cognitive function, growth, and overall health [19]. The increase in saturated fatty acids and the higher *n*-6 ratio in obese mothers are often related to dietary patterns, including a lower intake of ALA and DHA, as well as an overabundance of omega-6 fatty acids in the diet. These dietary imbalances may contribute to the altered composition of fatty acids in breast milk and may negatively affect the infant’s neurological development, as omega-3 fatty acids are crucial for brain function and development.

Maternal diet plays a critical role in shaping the fatty acid profile of breast milk, particularly the levels of DHA, ALA, and AA. Studies have shown that a poor maternal diet, especially one low in omega-3-rich foods like fatty fish, seeds, and nuts, can lead to reduced levels of these essential fatty acids in breast milk. This is particularly concerning for obese mothers, whose dietary patterns are often characterized by higher consumption of processed foods rich in omega-6 fatty acids and lower intake of omega-3s. As a result, the milk of obese mothers tends to have lower levels of ALA, DHA, and AA compared to that of mothers with a normal BMI [21].

Furthermore, obesity is associated with metabolic and hormonal changes that may influence fat metabolism and the synthesis of fatty acids. For instance, altered levels of adipokines, such as leptin and adiponectin, which are higher in obese mothers, can affect the transport and secretion of fatty acids into breast milk. These hormonal imbalances may contribute to the lower levels of essential fatty acids found in the milk of obese mothers, thereby impacting the overall quality of breast milk.

In this context, the research by Binder and colleagues emphasizes the positive impact of maternal protein intake, which is associated with higher levels of protein, fat, and energy in breast milk, as well as increased adiponectin and leptin levels—both crucial for the metabolic health of the infant [22].

Similarly, iodine deficiency is strongly linked to thyroid dysfunction and cognitive impairments, highlighting the need for adequate maternal iodine intake during pregnancy and lactation [26]. Interestingly, our review also reveals that specific dietary habits can enhance infant health outcomes. For instance, higher maternal protein intake is associated with improved breast milk quality, supporting better infant growth and neurodevelopment, particularly for preterm infants [33].

However, our findings also draw attention to the risks posed by certain dietary habits, highlighting the importance of avoiding dietary contaminants to ensure optimal nutrition for both mother and baby. The consumption of contaminated foods—such as pork meat, soft drinks, sweets, and wine—can lead to the presence of toxins like ochratoxin A in breast milk, which may negatively impact infant health [34].

An important finding from this review concerns the impact of maternal smoking on both breast milk composition and infant health outcomes. The studies highlight that smoking during pregnancy and breastfeeding significantly alters key bioactive compounds in breast milk, such as osteopontin, leptin, and mercury. These findings underscore the importance of public health initiatives aimed at smoking cessation during pregnancy and breastfeeding, and the need for further research to better understand the long-term consequences of maternal smoking on both breast milk quality and infant health [36,37,38].

This systematic review highlights several strengths, particularly in identifying critical gaps in the current evidence. While extensive research has examined maternal diet’s effects on breast milk composition and infant health, no studies were found that explored the role of maternal physical activity in this context, despite it being explicitly included in the search strategy. This lack of evidence is particularly striking given that physical activity was explicitly included in the search strategy. Moreover, this absence reveals a significant gap in the literature, as physical activity is known to benefit maternal health [47], but its impact on breast milk and infant outcomes remains largely unexplored. Additionally, the review shows that most existing studies focus on either maternal lifestyle and breast milk composition or the link between breast milk composition and infant health, rarely addressing the full interaction of all three factors. This systematic review highlights several strengths, particularly in identifying critical gaps in the current evidence. While extensive research has examined the effects of maternal diet on breast milk composition and infant health, no studies were found that investigated the role of maternal physical activity in this context, despite it being explicitly included in the search strategy. This absence of evidence is particularly striking, given that physical activity was clearly part of the search terms. Furthermore, this gap underscores a significant limitation in the literature, as physical activity is well established as benefiting maternal health [47,48], yet its impact on breast milk composition and infant health outcomes remains largely unexplored.

Despite the comprehensive scope of this review, several limitations must be considered. One significant limitation is the heterogeneity across the studies in terms of sample sizes, methodologies, and geographical locations, which complicates the generalization of findings to diverse populations. For instance, women in the included studies were at different stages of pregnancy and the infant health outcomes were assessed at various stages of development, which may contribute to inconsistencies in the results. Second, limitations pertaining to the methodological challenges in studying human breast milk (i.e., different sampling methods, biases in detection technologies), particularly its microbiota and nutritional composition, may compromise the reproducibility and comparability of findings.

The third limitation of this review lies in the methodological inconsistencies observed in the assessment of maternal dietary patterns and nutrient intake. Indeed, the lack of standardized approaches for analyzing key components such as fatty acids, micronutrients, and bioactive compounds may compromise the accuracy and reliability of nutrient assessments. The fourth limitation is the predominant reliance on observational data in many of the included studies, which constrains the ability to establish causality. Although clear associations between maternal nutrition and infant health outcomes were identified, the potential influence of confounding factors—such as socioeconomic status, pre-existing health conditions, and environmental exposures—was not consistently addressed. This limitation highlights the need for future studies employing robust experimental designs to better isolate the effects of maternal nutrition from these confounding variables.

## 5. Conclusions

This review emphasizes the vital role of maternal nutrition in influencing breast milk composition and infant health. The evidence highlights the need for targeted nutritional policies and educational programs, especially for populations at risk of nutrient deficiencies. Key nutrients are crucial for infant brain development and overall health, underscoring the importance of public health interventions to ensure proper maternal nutrition during pregnancy and breastfeeding. Additionally, socio-economic factors, food accessibility, and cultural practices must be addressed through tailored strategies. While physical activity and smoking cessation are recognized as important components of maternal health, their direct impact on breast milk composition was not explored in the studies included in this review. Integrating these lifestyle factors into materna health policies, alongside nutritional support, can further improve maternal and infant health outcomes. These efforts are crucial for empowering mothers to make informed dietary choices, reducing health disparities, and promoting better health for future generations.

## Figures and Tables

**Table 1:** Characteristics of studies investigating the effects of dietary and nutritional factors on breast milk and infant health.

**Author**	**Year**	Country	Study Population	Lifestyle Factor	Outcome	References
de la Garza Puentes et al.	2019	Spain	78 pregnant women and 78 infants followed at 6, 18, and 36 months	Maternal obesity	Proper neonatal growth and development, neurological development, and prevention of future non-communicable diseases	[19]
Tekin-Guler et al.	2023	Turkey	40 pregnant women and 40 infants at 2 months	Maternal obesity	Proper neonatal growth and development	[20]
Nyuar et al.	2010	Sudan	60 postpartum women and 60 infants	Lack of DHA-rich foods	Neuro-visual development	[21]
Binder et al.	2023	Austria	136 breastfeeding women and 136 infants	Maternal obesity	Proper nervous system growth and development	[22]
Bahreynian et al.	2023	Iran	215 pregnant women and 215 infants (birth to 1 year)	Incorrect fatty acid intake	Proper neonatal growth and development	[23]
Hoppu et al.	2005	Finland	34 pregnant women with atopic disease and 34 infants (birth to 1 year)	Vitamin C intake	Atopic diseases and allergies in infants	[24]
Han et al.	2021	China	264 breastfeeding women and 264 infants	Selenium intake	Infant growth and development	[25]
Næss et al.	2023	Norway	137 pregnant women and 113 infants (3 months to 11 months)	Iodine intake	Thyroid function and cognitive development	[26]
Stinca et al.	2017	Morocco	245 pregnant women and 239 breastfeeding women, and 239 infants	Low-iodine diet	Hypothyroidism and cognitive disorders	[27]
Finkelstein et al.	2013	Peru	59 pregnant women and 59 infants at 3 months	Iron supplementation	Adequate growth	[28]
Warstedt et al.	2016	Sweden	95 pregnant women and 95 infants (3 months to 2 years)	Omega-3 supplementation	Immunoglobulin-associated diseases	[29]
Dunstan et al.	2007	Australia	98 pregnant women and 98 infants at 1 year	Fish oil supplementation	Cognitive development and eye–hand coordination	[30]
Bottcher et al.	2008	Sweden	109 women (36th week of pregnancy to childbirth) and 109 infants (birth to 2 years)	Probiotic supplementation with Lactobacillus reuteri	Eczema and allergies	[31]
Boyle et al.	2011	Australia	250 women (36th week of pregnancy) and 250 infants (up to 1 year)	Probiotic supplementation with Lactobacillus rhamnosus	Eczema and allergies	[32]
Lutter et al.	2018	United States	Pregnant women and infants aged 7 to 12 months	Egg consumption	Cognitive development, memory, and adequate growth	[33]
Biasucci et al.	2011	Italy	130 pregnant women and 130 infants	Consumption of soft drinks and foods containing ochratoxin A	Increased risk of cancer, kidney diseases, and neurological disorders	[34]
Henning et al.	2022	United States	10 mothers 6 months postpartum and 10 infants	Pomegranate juice intake	Intestinal microbiota composition and optimal health	[35]

**Table 2:** Characteristics of studies investigating the effects of smoking habits on breast milk and infant health.

Author	Year	Country	Study Population	Lifestyle Factor	Outcome	References
Aksan et al.	2021	Turkey	85 breastfeeding mothers and 85 infants	Maternal smoking	Altered immune system development, increased risk of infections, impaired growth	[36]
Yalçin et al.	2010	Turkey	67 mothers (20 days postpartum) and 44 exclusively breastfed infants (5 months)	Passive smoking	Normal growth and development	[37]
Ozkan et al.	2005	Turkey	44 breastfeeding mothers and 44 infants (1 week postpartum)	Smoking at least 5 cigarettes daily	Risk of low birth weight and impaired fetal growth	[38]

## Data Availability

No new data were created or analyzed in this study. Data sharing is not applicable to this article.

## Supplementary Materials

The following supporting information can be downloaded at: https://www.mdpi.com/article/10.3390/nu17010062/s1, Table S1: PRISMA checklist.
